# Preoperative versus postoperative chemo-radiotherapy for locally advanced gastric cancer: a multicenter propensity score-matched analysis

**DOI:** 10.1186/s12885-022-09297-7

**Published:** 2022-02-26

**Authors:** Ning Li, Xiaoyong Xiang, Dongbin Zhao, Xin Wang, Yuan Tang, Yihebali Chi, Lin Yang, Liming Jiang, Jun Jiang, Jinming Shi, Wenyang Liu, Hui Fang, Yu Tang, Bo Chen, Ningning Lu, Hao Jing, Shunan Qi, Shulian Wang, Yueping Liu, Yongwen Song, Yexiong Li, Liyuan Zhang, Jing Jin

**Affiliations:** 1 Department of Radiation Oncology, National Cancer Center/National Clinical Research Center for Cancer/Cancer Hospital, Chinese Academy of Medical Sciences and Peking Union Medical College, Beijing, 100021 China; 2 Department of Radiation Oncology, National Cancer Center/National Clinical Research Center for Cancer/Cancer Hospital & Shenzhen Hospital, Chinese Academy of Medical Sciences and Peking Union Medical College, Shenzhen, 518116 China; 3 Department of Abdominal Surgical Oncology, National Cancer Center/National Clinical Research Center for Cancer/Cancer Hospital, Chinese Academy of Medical Sciences and Peking Union Medical College, Beijing, 100021 China; 4 Department of Medical Oncology, National Cancer Center/National Clinical Research Center for Cancer/Cancer Hospital, Chinese Academy of Medical Sciences and Peking Union Medical College, Beijing, 100021 China; 5 Department of Radiology, National Cancer Center/National Clinical Research Center for Cancer/Cancer Hospital, Chinese Academy of Medical Sciences and Peking Union Medical College, Beijing, 100021 China; 6 Department of Radiation Oncology, Institute of Radiation Oncology, the Second Affiliated Hospital of Soochow University/Suzhou Key Laboratory for Radiation Oncology, Soochow University, Suzhou, 215004 Jiangsu China; 7 State Key Laboratory of Molecular Oncology, Department of Radiation Oncology, National Cancer Center/Cancer Hospital, Chinese Academy of Medical Sciences, Peking Union Medical College, Beijing, 100021 China

**Keywords:** Gastric cancer, Preoperative chemo-radiotherapy, Postoperative chemo-radiotherapy, Long-term outcome

## Abstract

**Background:**

Peri-operative chemo-radiotherapyplayed important rolein locally advanced gastric cancer. Whether preoperative strategy can improve the long-term prognosis compared with postoperative treatment is unclear. The study purpose to compare oncologic outcomes in locally advanced gastric cancer patients treated with preoperative chemo-radiotherapy (pre-CRT) and postoperative chemo-radiotherapy (post-CRT).

**Methods:**

From January 2009 to April 2019, 222 patients from 2 centers with stage T3/4 and/or N positive gastric cancer who received pre-CRT and post-CRT were included. After propensity score matching (PSM), comparisons of local regional control (LC), distant metastasis-free survival (DMFS), disease-free survival (DFS) and overall survival (OS) were performed using Kaplan-Meier analysis and log-rank test between pre- and post-CRT groups.

**Results:**

The median follow-up period was 30 months. 120 matched cases were generated for analysis. Three-year LC, DMFS, DFS and OS for pre- vs. post-CRT groups were 93.8% vs. 97.2% (*p* = 0.244), 78.7% vs. 65.7% (*p* = 0.017), 74.9% vs. 65.3% (*p* = 0.042) and 74.4% vs. 61.2% (*p* = 0.055), respectively. Pre-CRT were significantly associated with DFS in uni- and multi-variate analysis.

**Conclusion:**

Preoperative CRT showed advantages of oncologic outcome compared with postoperative CRT.

**Trial registration:**

ClinicalTrial.gov NCT01291407, NCT03427684 and NCT04062058, date of registration: Feb 8, 2011.

## Background

In China, 6.791 million new cases and 498 thousand deaths of gastric cancer every year, and 70.8% of newly diagnosed patients were locally advanced stage [1, 2]. The crucial role of peri-operative chemo-radiotherapy in locally advanced gastric cancer have been concluded by studies [3–9]. Postoperative radiotherapy based on pathological stages, while preoperative radiotherapy has the advantages of down staging and lower rate of severe adverse events.

However, whether preoperative strategy could improve the prognosis compared with postoperative treatment is unclear [10, 11]. The purpose of this study was to compare long-term outcomes in locally advanced gastric cancer patients after preoperative chemo-radiotherapy (pre-CRT) and postoperative chemo-radiotherapy (post-CRT).

## Methods

### Patients and eligibility

From January 2009 to April 2019, patients from 2 centers with locally advanced gastric adeno-carcinoma who received pre-CRT or post-CRT were included. The inclusion criteria were as follows: 18–75 years old, male or female; stage T3–4 and/or N+ gastric cancer without distant metastasis; Karnofsky score ≥ 70; normal haematology examination. For pre-CRT patients, radiological examinations, including CT, MRI with or without PETCT, and gastroscopy should be performed for clinical TNM stage and pathology diagnosis. For post-CRT patients, pathology stage should be confirmed by post-operative histo-pathological results. All patients signed informed consent forms.

### Treatment regimens

Pre-CRT patients were initially treated with radiotherapy concurrent with S-1. Three weeks after radiotherapy, patients were given neo-adjuvant chemotherapy with oxaliplatin and S-1 (SOX). Pre-operative imaging evaluation was performed 21 days after neo-adjuvant treatment. The surgical procedures were determined based on multidisciplinary team (MDT) discussion. In-operable patients continued with 3 cycles of chemotherapy, and the chemotherapy regimen was not specified. The patients in post-CRT group received radiotherapy concurrent chemotherapy, which with S-1 or capecitabine regimen, after radical resection. D2 resection and adjuvant chemotherapy was recommended for entire cohort. And pre- or post-operative radiotherapy dose was prescribed as 45Gy, with intensity modulated radiotherapy (IMRT) or volumetric modulated arc radiotherapy (VMAT) technique. ITV was included in the margin of PTV. 4DCT or abdominal compression devices was not mandatory for CT-sim or treatment.

### Evaluation and endpoints

The preoperative TNM stage was evaluated via gastric MRI, gastroscopy, endoscopic ultrasonography and CT images of thoracic, abdominal and pelvic. Diagnostic laparoscopy and PETCT scans were not mandatory. Surgical resection specimens were subjected to a extensive evaluation of primary lesions and lymph nodes.

Follow-up was scheduled at 3-month intervals for the first 2 years and then at 6-month intervals until 5 years. Diagnostic evaluations were performed using CT of the chest and abdomen and MRI or gastroscopy only if necessary. The primary endpoint was disease-free survival (DFS), defined as the interval from the date of the surgery for post-CRT group or the first pre-CRT to the date of recurrence or death from any cause. The secondary endpoints were overall survival (OS), local control (LC) and distant metastasis free survival (DMFS).

### Statistical analysis

Since patients were not randomly assigned to either treatment group due to the retrospective nature of the analysis, propensity score matching (PSM) was used to determine the independent impact of treatment modality on long-term oncologic outcomes. First, logistic regression using these variables was performed to obtain the propensity score for each patient (defined as the probability to be assigned to pre- or post-CRT group according to the individual profile of these covariates). Then, patients in each group were matched according to the calculated propensity scores using a k nearest neighbours (KNN) algorithm with a threshold of c ≤ 0.05. After matching, Kaplan-Meier analysis for LC, DMFS, DFS and OS were performed and compared between two groups using log-rank test.

Statistical analysis was performed by the SPSS Version 22 software (IBM Corporation, Armonk, NY, USA). A two-sided *p*-value of < 0.05 was considered significant.

The Kaplan-Meier method was used to analyse the survival rate using R software (R Foundation for Statistical Computing, Vienna, Austria).

## Results

### Clinical characteristics

Two hundred and twenty two patients were enrolled, and the follow-up rate was 100%. In total, 79.3% were male patients. The median age was 60 (27–75) years. 89.6 and 84.2% of patients was T3/4 lesions and clinical N positive, respectively. Table 1 summarizes the patients’ baseline characteristics for each group, indicating relevant differences between the two. Patients in pre-CRT group significantly had a greater frequency of proximal segment gastric cancer, poorly differentiated pathological type, clinical T3/4 and N1/2 gastric cancer than in post-CRT group. Median dose of radiotherapy delivered was 45Gy(41.4-45Gy) and 45Gy(39.6-45Gy) in pre- and post-CRT group, respectively.

**Table 1:** Patient characteristics by treatment group before and after PSM

	Total	Entire cohort			PSM cohort		
	(*n* = 222, %)	pre-CRT (*n* = 92, %)	post-CRT (*n* = 130, %)	*p*	pre-CRT (*n* = 60, %)	post-CRT (*n* = 60, %)	*p*
Sex							
Male	169 (76.1)	73 (79.3)	96 (73.8)	0.344	48 (80.0)	47 (78.3)	0.822
Female	53 (23.9)	19 (20.7)	34 (26.2)		12 (20.0)	13 (21.7)	
Median age	60 (27–75)	61 (35–75)	59 (27–75)	0.657	60 (35–73)	60 (31–75)	0.817
Segment							
Proximal	87 (39.2)	57 (62.0)	30 (23.1)	0.000	31 (51.7)	25 (41.7)	0.171
Body	54 (24.3)	12 (13.0)	42 (32.3)		11 (18.3)	20 (33.3)	
Distal	81 (36.5)	23 (25.0)	58 (44.6)		18 (30.0)	15 (25.0)	
Pathological type							0.103
Well differentiated	1 (0.4)	1 (1.1)	0 (0.0)	0.000	0 (0.0)	0 (0.0)	
Moderate differentiated	60 (27.0)	14 (15.2)	46 (35.4)		14 (23.3)	19 (31.7)	
Poorly differentiated	124 (55.9)	67 (72.8)	57 (43.8)		41 (68.3)	31 (51.7)	
Mucinous adenocarcinoma	12 (5.4)	2 (2.2)	10 (7.7)		2 (0.3)	5 (8.3)	
Signet ring cell carcinoma	20 (9.0)	7 (7.6)	13 (10.0)		3 (0.5)	5 (8.3)	
Unknown	5 (2.3)	1 (1.1)	4 (3.1)		0 (0.0)	0 (0.0)	
T stage^a^				0.000			0.691
T1	4 (1.8)	0 (0.0)	4 (3.1)		0 (0.0)	0 (0.0)	
T2	19 (8.6)	2 (2.2)	17 (13.1)		2 (3.3)	4 (6.7)	
T3	106 (47.7)	37 (40.2)	69 (53.1)		29 (48.3)	29 (48.3)	
T4	93 (41.9)	53 (57.6)	40 (30.8)		29 (48.3)	27 (45.0)	
N stage^a^							
N0	35 (15.8)	11 (11.6)	24 (18.5)	0.000	10 (16.7)	12 (20.0)	0.068
N1	49 (22.0)	27 (29.3)	22 (16.9)		17 (28.3)	12 (20.0)	
N2	65 (29.3)	36 (39.1)	29 (22.3)		21 (35.0)	12 (20.0)	
N3	73 (32.9)	18 (19.6)	55 (42.3)		12 (20.0)	24 (40.0)	
Surgical procedure							
D1	35 (15.8)	9 (9.8)	26 (20.0)	0.000	9 (15.0)	12 (20.0)	0.332
D1+	47 (21.2)	17 (18.5)	30 (23.1)		12 (20.0)	17 (28.3)	
D2	118 (53.1)	44 (47.8)	74 (56.9)		39 (65.0)	31 (51.7)	
No operation	22 (9.9)	22 (23.9)	–				
Peri-operative chemo.							
Yes	194 (87.7)	75(81.5)	119(91.5)	0.049	55(91.7)	56(93.3)	0.841
No	28 (12.6)	17(18.5)	11(8.5)		5(8.3)	4(6.7)	

^a^T and N stage of pre-CRT group were clinical staging

In pre-CRT group, the median duration between neo-adjuvant treatment and surgery was 52 (14–174) days. Twenty-two patients (23.9%) did not undergo a further surgical procedure because of disease progression or other personal reasons. Among these patients, 17 had distant metastasis (4 peritoneal, 4 liver, 2 para-aortic lymph nodes, 2 ovarian and 1 lung metastasis, and 5 unknown), 5 abandoned the operation due to personal reasons or other unknown reasons. Among the 70 resected patients, the rate of downstaging, ypN0 and pathologic complete response (pCR) rate was 64.1% (*n* = 59), 50.0% (*n* = 46) and 15.2% (*n* = 14) respectively. In post-CRT group, 119 patients (91.5%) underwent adjuvant chemotherapy, which was more than 81.5% in pre-CRT group.

### Entire cohort prior to propensity score matching

**Fig. 1:**
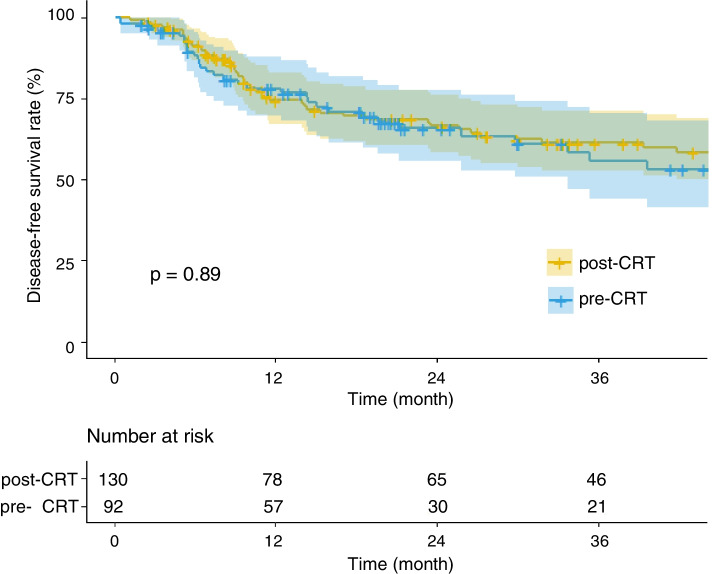
Kaplan-Meier plots for disease-free survival (DFS) for entire cohort

The median follow-up for survivors was 30 (range: 8–84) months in pre-CRT group and 39 (range: 6–90) months in post-CRT group, respectively. There were no significant differences in clinical outcomes between the two groups before PSM analysis (Table 2 and Fig. 1).

**Table 2:** Long-term outcome of pre- and post-CRT Group before and after PSM

	Entire cohort (*n* = 222)			PSM cohort (*n* = 120)		
	Pre-CRT	Post-CRT	*p*	Pre-CRT	Post-CRT	*p*
3-year LC	90.6%	95.6%	0.056	93.8%	97.2%	0.244
3-year DMFS	59.6%	65.7%	0.922	78.7%	65.7%	0.017
3-year DFS	56.3%	61.2%	0.998	74.9%	65.3%	0.042
3-year OS	62.4%	64.5%	0.668	74.4%	61.2%	0.055

Table 3 presents the results of uni- and multi-variate Cox proportional hazards models for DFS. Clinical T/N stage and surgical procedure were associated with DFS in the univariate analysis and were included in the multivariate model. By multivariate analysis, surgical procedure was associated with improved DFS (*p* = 0.001) Table 4.

**Table 3:** Univariate and multivariate Cox proportional hazards models for DFS

Variable	Entire cohort Events/total 81/222		PSM cohort Events/total 36/120	
	HR (95%CI)	*p*	HR (95%CI)	*p*
*Univariate*				
Sex		0.240		0.147
Male	1.335 (0.832–2.140)		1.024 (0.465–2.253)	
Female	1		1	
Segment		0.975		0.422
Proximal	1		1	
Body	0.990(0.840–1.167)		0.840(0.559–1.263)	
Distal	0.951(0.723–1.098)		0.845(0.650–1.099)	
Pathological type		0.123		0.373
Well differentiated	1		–	
Moderate differentiated	1.000(0.561–1.652)		1	
Poorly differentiated	1.001(0.652–1.623)		1.465(0.933–2.302)	
Mucinous adenocarcinoma	1.986(0.968–3.632)		1.235(0.873–1.748)	
Signet ring cell carcinoma	1.020(0.862–1.774)		1.149(0.686–1.603)	
T stage		0.046		0.508
T1	1		–	
T2	1.057(0.656–1.703)		1	
T3	1.184(0.783–1.805)		1.489(0.430–6.744)	
T4	1.357(0.856–2.013)		1.815(0.657–5.017)	
N stage		0.002		0.022
N0	1		1	
N1	1.170(0.505–2.710)		1.186(0.672–2.092)	
N2	1.234(0.778–1.655)		1.279(0.781–2.181)	
N3	1.338(1.061–1.689)		1.307(0.962–1.777)	
Surgical procedure		0.001		0.788
D1	1.230(0.685–2.207)		1.423(0.532–3.805)	
D1+	1.091(0.569–2.091)		1.119(0.524–2.392)	
D2	1		1	
No operation	1.213(1.106–1.331)		–	
Peri-operative chemo.		0.988		0.369
Yes	1		1	
No	1.005(0.630–1.807)		1.202(0.754–2.244)	
CRT timing		0.998		0.042
Pre-	1		1	
Post-	1.000(0.636–1.575)		2.127(1.010–4.420)	
*Multivariate*				
T stage		0.223		
T1	1			
T2	1.149(0.712–1.889)			
T3	1.184(0.783–1.905)			
T4	1.151(0.479–1.439)			
N stage		0.336		0.578
N0	1		1	
N1	1.772(0.946–1.812)		1.379(0.661–3.370)	
N2	2.059(1.039–2.211)		1.648(0.872–4.549)	
N3	2.565(1.526–2.699)		1.307(0.806–7.008)	
Surgical procedure		0.001		
D1	1.520(0.997–3.237)			
D1+	1.290(1.047–1.446)			
D2	1			
No operation	2.213(0.881–5.440)			
CRT timing				0.038
Pre-			1	
Post-			2.114(1.291–8.140)

**Table 4:** Long-term outcomes summary of peri-operative radiotherapy from randomized trial in gastric cancer

	Study	Randomization design	Inclusion criteria	OS of RT group	DFS of RT group	Comments
Pre-CRT	CAMS/PUMC [8]	RT + S vs. S	Local advanced gastric cancer.	5y-OS 30.1% 10y-OS 20.26%	–	Proportion of D2 resection: 40%
	CROSS [12]	CRT + S vs. S	Oesophageal or junctional cancer; T1–3N0–1M0(UICC 6th edition).	1y-OS 81% 2y-OS 67% 3y-OS 58% 5y-OS 47%	1y-PFS 71% 2y-PFS 60% 3y-PFS 51% 5y-PFS 44%	Proportion of EGJ 22–26%
	POET [13]	CT + CRT + S vs. CT + S	Adenocarcinoma of EGJ; T3-T4(UICC 5th edition).	3y-OS 46.7% 5y-OS 39.5%	–	–
Post-CRT	INT-0116 [14]	S + CRT vs. S	Adenocarcinoma of the stomach or EGJ; IB ~ IVM0.	3y-OS 50%	3y-RFS 48%	Proportion of D2, D1, D0 resection: 10, 36, 54%; Proportion of EGJ 7.0%
	ARTIST [15]	S + XP + CRT + XP vs. S + XP	Gastric cancer; IB-IV(M0) (AJCC 6th edition); D2 resection.	5y-OS 75%	3y-DFS 78%	–
	CALGB 80101 [16]	S + CT + CRT + CT ECF vs. FU/LV CRT with FU	Gastric cancer /EGJ; IB-IV(M0) (AJCC 6th edition).	5y-OS 44%	5y-DFS 37% vs. 39% (FU/ LV: ECF)	Proportion of EGJ 22%
	CRITICS [17]	ECC + S + ECC ECC + S + CRT	Gastric cancer/EGJ; IB-IVa (AJCC 6th edition).	5y-OS 42%	5-year EFS 38%	Proportion of D2 + D3 < 10%; Proportion of EGJ 17.1%
	ARTIST II [18]	S + CRT S + S1 S + SOX	Gastric cancer; Stage II-III; N+; D2 resection.	–	3y-DFS 73%	–

*RT* radiotherapy, *CRT* chemo-radiotherapy. *S* surgery, *EGJ* esophagogastric junction, *OS* overall survival, *DFS* disease-free survival, *EFS* event-free survival, *RFS* relapse-free survival, *PFS* progress-free survival

### Propensity score-matched cohort

**Fig. 2:**
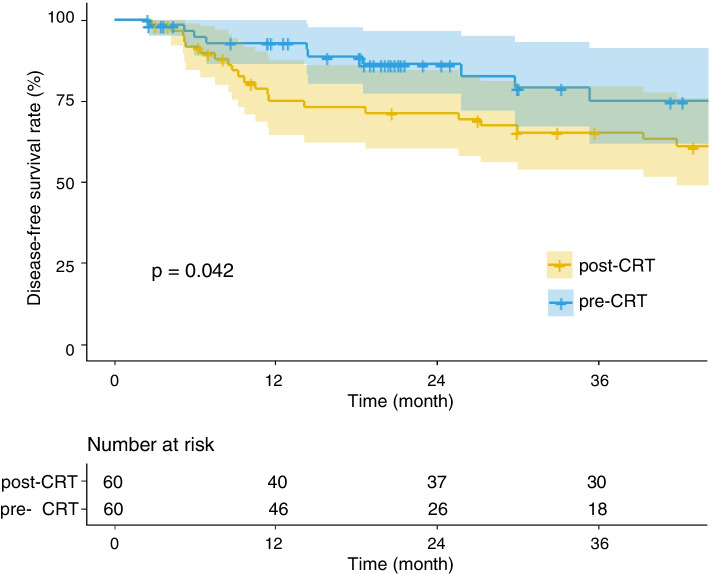
Kaplan-Meier plots for disease-free survival (DFS) after PSM

Propensity score matching resulted in 60 matched pairs (pre-: post-CRT = 1:1), for a total of 120 patients. Patient and tumour characteristics were not significantly different between two groups of matched pairs (Table 1), indicating that the matching procedure worked well. After PSM, the Pre-CRT group patients resulted in superior 3-year DFS (74.9% vs.65.3%, *p* = 0.042; Fig. 2) and DMFS rate (78.7% vs. 65.7%, *p* = 0.017) to those in post-CRT group. The pre-CRT group showed a better 3-year OS trend (74.4% vs. 61.2%, *p* = 0.055) as compared with post-CRT group. No LC difference between these two groups was observed (93.8% vs. 97.2%, *p* = 0.244) (Table 2).

Clinical N stage and pre-CRT were significantly associated with DFS in the univariate analysis. And pre-CRT remained significant in the multivariate model (*p* = 0.038) (Table 2) in PSM cohort.

## Discussion

The optimal strategy for locally advanced gastric cancer is peri-operative comprehensive treatment, including peri-operative chemotherapy, radiotherapy and novel molecular agents. To our knowledge, few studies have explored to compare the long-term outcomes of preoperative with postoperative chemo-radiotherapy in gastric cancer with PSM method. The survival analysis after PSM indicated that DFS rate of pre-CRT group was significant higher than that of post-CRT. And the pre-CRT group showed a trend towards to better 3-year OS.

Radiotherapy plays an important role in the comprehensive treatment of locally advanced gastric cancer. Seyedin et al. analyzed the prognosis of 21,472 patients with stage I-IV gastric cancer in SEER database. For patients with stage II, III, or IV, those treated with radiotherapy had the best outcome compared with the other treatment modalities [12]. The study based on 21,447 cases of gastric cancer from the NCDB database showed that the use of RT in addition to chemotherapy was associated with a significant OS advantage [13]. In randomized studies of postoperative radiotherapy, although the series of ARTIST studies did not obtain positive results, INT0116 and CRITICS studies suggested that postoperative radiotherapy was effective for patients with specific treatment modality and disease stage [14, 15, 19, 20]. Published clinical studies concerning neo-adjuvant treatment showed that preoperative CRT could improve the pCR rate and long-term outcomes [9]. The phase 3 randomized controlled study from our centre compared the prognosis of preoperative radiotherapy with that of surgery alone. The 5- and 10-year OS rates in the preoperative radiotherapy cohort were significantly better [8]. The CROSS study conducted similar results [5]. And our previous study reported the prognosis of preoperative CRT compared with that of preoperative chemotherapy. The 2-year DFS and LRFS rates of CRT group were better than preoperative chemotherapy [21]. Therefore, both the analysis based on big data and prospective randomized studies confirmed the value of radiotherapy. And radiotherapy is recommended as standard treatment for local advanced gastric cancer in NCCN and ESMO guidelines.

Local advanced gastric cancer is eligible for either pre- or post-operative CRT. However, at present, there is no large sample prospective randomized controlled study comparing these two strategies. In some pooled analysis studies, which compared pre- with post-CRT, results were inconsistent. Wong reviewed 16 randomized controlled studies, 3 meta-analyses and 1 practice guideline of preoperative CRT and postoperative CRT for gastric cancer [22]. They concluded that preoperative CRT is a very promising treatment strategy for local advanced gastric cancer. However, the results from SEER database study showed that for stage II patients the death hazard risk of treatment with adjuvant radiotherapy was the lowest. For patients with stage III-IV, there was no significant difference in death hazard risk between the pre- or post-operative radiotherapy strategy [12]. In the Afsaneh study, the results were similar. Twenty-one thousand four hundred and forty-seven cases of gastric cancer included in the NCDB database were divided into three groups: perioperative chemotherapy + operation group, perioperative chemotherapy + operation + adjuvant radiotherapy group and neo-adjuvant radiotherapy + operation + chemotherapy group. The results showed that the overall survival rate of the adjuvant radiotherapy group was the best (*P* < 0.001) [23]. Our study compared the long-term prognosis of pre- and post-operative radiotherapy patients with PSM statistical method, which could minimize the selection bias between two groups. The results confirmed that preoperative radiotherapy had more advantages in the long-term prognosis.

The advantages of the preoperative treatment of gastric cancer include an improved R0 resection rate by down-staging, tolerable toxicities and a good long-term prognosis. However, the accuracy of the preoperative clinical staging of gastric cancer, especially the diagnosis of peritoneal metastasis, is challenging the clinical practice. In studies reported by surgeons, the incidence of intra-operative observed peritoneal metastasis could be as high as 30% in imaging diagnosed clinical M0 stage patients [16]. Patients with underestimated staging will progress during preoperative radiotherapy. Therefore, underestimating the clinical stage might be major issue, which may affect the overall prognosis of preoperative treatment modality. In the uni- and multi-variate factor analysis of our study, we found that the surgery was a good prognostic factor for long-term outcomes. And the main reason that patients did not receive surgery was disease progression, most likely caused by the underestimation of staging. After PSM, these patients without operation in pre-CRT group due to paired un-matching was excluded for further survival analysis. This might be the main cause of better DFS in pre-CRT group. Therefore, the accuracy of clinical staging before initial treatment is very important in the subsequent randomized controlled study and clinical practice.

In recent years, total neoadjuvant treatment has become a topic of high interest in the treatment of GI cancer, which can lead to downstaging and pCR [17]. The expected greater opportunity for delivering high-dose chemotherapy in the preoperative setting could theoretically improve the rate of R0 resection of the cancer, and thus increase relapse-free survival. This hypotheses was demonstrated in Stahl’s study. In this study, compared with preoperative chemotherapy, higher pCR, ypN0 and better OS tendency was achieved by chemo-radiotherapy, although the study recruited only 126 patients due to a slow recruiting speed.

There were some limitations in this study. First, the chemotherapy regimen and cycles were not detailed enough to evaluate the perioperative chemotherapy intensity of all patients, which might have influenced the long-term prognosis. Second, gastric cancer is highly heterogeneous. There were limited clinical and pathological factors that might be related to prognosis that were analysed in this study. Third, although the data come from two centers, the sample size was still not large enough, which might lead to biased results.

In conclusion, preoperative chemo-radiotherapy may have better long-term outcomes for locally advanced gastric cancer, compared with post-operative chemo-radiotherapy. Despite these encouraging results, further prospective randomized studies should be conducted.

## Data Availability

The datasets used and/or analyzed during the current study are available from the corresponding author upon reasonable request.

## Abbreviations

pre-CRT: chemo-radiotherapy
post-CRT: postoperative chemo-radiotherapy
MDT: multidisciplinary team
IMRT: intensity modulated radiotherapy
VMAT: volumetric modulated arc radiotherapy
DFS: disease-free survival
OS: overall survival
LC: local control
DMFS: distant metastasis free survival
PSM: propensity score matching
KNN: k nearest neighbours
pCR: pathologic complete response
